# Perinatal Mortality Magnitude, Determinants and Causes in West Gojam: Population-Based Nested Case-Control Study

**DOI:** 10.1371/journal.pone.0159390

**Published:** 2016-07-28

**Authors:** Robel Yirgu, Mitike Molla, Lynn Sibley, Abebe Gebremariam

**Affiliations:** 1 Department of Reproductive Health and Health Service Management, School of Public Health, College of Health Sciences, Addis Ababa University, Addis Ababa, Ethiopia; 2 Nell Hodgson Woodruff School of Nursing and Rollins School of Public Health, Emory University, Atlanta, Georgia, United States of America; 3 Maternal and Newborn Health in Ethiopia Partnership project, Emory University, Addis Ababa, Ethiopia; Centre Hospitalier Universitaire Vaudois, FRANCE

## Abstract

**Introduction:**

In Ethiopia, even if a significant reduction in child mortality is recorded recently, perinatal mortality rate is still very high. This study assessed the magnitude, determinants and causes of perinatal death in West Gojam zone, Ethiopia.

**Methods and materials:**

A nested case control study was conducted on 102 cases (mothers who lost their newborns for perinatal death) and 204 controls (mothers who had live infants in the same year) among a cohort of 4097 pregnant mothers in three districts of the West Gojam zone, from Feb 2011 to Mar 2012. Logistic regression models were used to identify the independent determinant factors for perinatal mortality. The World Health Organization verbal autopsy instrument for neonatal death was used to collect mortality data and cause of death was assigned by a pediatrician and a neonatologist.

**Result:**

Perinatal mortality rate was 25.1(95% CI 20.3, 29.9) per 1000 live and stillbirths. Primiparous mothers had a higher risk of losing their newborn babies for perinatal death than mothers who gave birth to five or more children (AOR = 3.15, 95% CI 1.03–9.60). Babies who were born to women who had a previous history of losing their baby to perinatal death during their last pregnancy showed higher odds of perinatal death than their counterparts (AOR = 9.55, 95% CI 4.67–19.54). Preterm newborns were more at risk for perinatal death (AOR = 9.44, 95%CI 1.81–49.22) than term babies. Newborns who were born among a household of more than two had a lesser risk of dying during the perinatal period as compared to those who were born among a member of only two. Paradoxically, home delivery was found to protect against perinatal death (AOR = 0.07 95% CI, 0.02–0.24) in comparison to institutional delivery. Bacterial sepsis, birth asphyxia and obstructed labour were among the leading causes of perinatal death.

**Conclusion:**

Perinatal mortality rate remains considerably high, but proper maternal and child health care services can significantly decrease the burden.

## Introduction

Perinatal death has been shadowing the joy of bearing children for many parents in the history of human kind. Though there is a decline in child mortality, still perinatal mortality is considerably high in the developing world [1]. Globally, neonatal mortality accounts for 38% of all under five mortalities where 75% of the deaths took place during the first week of the neonatal period [2]. From the 6.3 million perinatal deaths (i.e. 3.3 million stillbirths and 3 million early neonatal deaths) that were estimated to occur annually, 98% took place in developing countries [1, 2].

Ethiopia, like most sub-Saharan Africa countries, is suffering from high perinatal mortality [1]. In 2011 the national average perinatal mortality rate was reported to be 46 per 1000 births [3]. In this report the Amhara regional state, where this study was conducted, had a perinatal mortality rate of 55 per thousand, which was the second to the highest perinatal mortality rates of all the regions in the country [3]. The WHO Country, Regional and Global estimates of perinatal mortality indicated that Ethiopia is among leading countries of the world as far as perinatal mortality rate is concerned [1].

Most of the contributing factors for perinatal mortality are health care service related. Studies showed that suboptimal antenatal, intrapartal and postnatal health care services are strongly associated with adverse pregnancy outcomes [4]. Age of the mother at pregnancy, maternal education, and wealth are some of the socioeconomic and demographic factors often associated with perinatal mortality [5]. In areas where mothers have access to quality health care services, congenital malformations, preterm birth and intra uterine growth restriction (IUGR) are the leading causes of perinatal death [6]. But in areas where there is low health care service coverage, the leading causes are asphyxia, neonatal tetanus, and neonatal infections [7, 8].

In Ethiopia, even if there is unacceptably high number of perinatal deaths, only little is known about its magnitude at sub-regional levels, also the determinants and causes of perinatal mortality is not well studied. Therefore, this study was conducted to estimate the magnitude, and identify determinants and causes of perinatal mortality in rural Amhara.

## Methods

### Study design and setting

We conducted a population-based nested case control study among a cohort of pregnant mothers in three districts of West Gojam zone (North Achefer, South Achefer and Mecha). The zone is located 500 kms away to the north of the capital city, Addis Ababa. Twenty four kebles (i.e. the smallest administrative unit) were selected from the three districts; 7 from North and South Achefer each and 10 from Mecha district. The selected three districts were among the highly populous districts of the zone with a total population count of 292,250 in Mecha, 155,863, in South Achefer and 173,211 in North Achefer districts [9]. Each kebele had one health post run by community Health Extension Workers (HEWs), there were two health centers in each district and Bahir Dar Hospital serves as a referral hospital for the people living in the three districts [9].

The cohort was established in mid 2010 by Maternal and Newborn Health in Ethiopia Partnership (MaNHEP) project in collaboration with Ethiopian Federal Ministry of Health, Emory University and Addis Ababa University. Pregnant mothers, in their third trimester, were enrolled in to the cohort after they were identified by trained community volunteers. Once in the cohort, mothers and their close family members (i.e. mothers, mother-in-laws, and husbands) received repeated training on care during pregnancy, labour and delivery by the volunteers. Following delivery they stayed in the cohort till they received postnatal care (PNC) mainly by HEWs [10]. Though the cohort was established in mid 2010 this study involved only pregnant mothers who gave birth between March 2011 and Feb 2012.

### Participants

Out of 4097 pregnant mothers who were followed in the cohort, all mothers (102) who lost their newborns for perinatal death were included in the study as cases. The controls were 204 mothers who gave birth to a live baby who at least survived the first 28 days after birth. The controls were randomly selected from the list of mothers with a known pregnancy outcome that were registered forming the sampling frame. To minimize the effect of geographic differences, controls were randomly selected from the gotes (i.e. smaller segment of a kebele) of the respective cases using the sampling frame which contained list of all mothers who gave birth in the three districts.

### Variables and data collection

Perinatal death was the dependent variable. The independent variables under the socio demographic category were age of the mother, marital status, educational status, occupation, size of the household, where the index neonates were not counted as members of the household and household wealth. Pregnancy, labour and delivery related variables such as, gestational age (calculated from the last menstrual period), birth spacing, place of delivery, parity, history of perinatal death, history of abortion (both spontaneous and medically induced termination of pregnancy before the 28^th^ week of gestation) were included.

Three high school complete female data collectors who were trained for 5 days, collected the data. Mothers who lost their newborns were interviewed, earliest forty days after death of the newborn to minimize recall bias. In addition we used female interviewers, so that mothers would be comfortable to discuss reproductive health matters that they may not be comfortable to discuss with men.

### Statistical methods

Data was entered using **EpiData** *version 3*.*1 statistical* software. After entry the data was exported to **SPSS** *version 19* statistical software for analysis. Perinatal, early neonatal and stillbirth rates were calculated. Bivariate analysis was conducted to measure the association between the dependent and individual independent variables. To control the effect of confounding variables multiple binary logistic regression models were used. Crude and adjusted OR with 95% CI were used to interpret findings of the bivariate and multivariate analysis.

A total of 15 dichotomous household asset variables were involved to generate wealth index using *Principal component analysis*. According to the index, households were divided into quintiles ranging from the poorest 20% to the richest 20%.

The cause of death assignment (CODA) was done by two physicians, a neonatologist and a pediatrician. In the process of CODA, the verbal autopsy (VA) data was reviewed and causes of death were assigned separately for every case. Then consensual diagnosis was reached after the two physicians discussed on their views concerning the causes of death for every case.

Finally the physicians gave codes to the identified causes of death according to the ICD-10 coding system.

### Ethical consideration

Ethical clearance was obtained from Addis Ababa University, College of Health Sciences, School of Public Health, Research and Ethics Committee. Prior to every interview the purpose of the study was explained to the participants and written consent was obtained. Mothers who lost their newborns were interviewed after forty days of culturally appropriate grieving period.

## Results

### Source population profile and perinatal mortality rate

A total of 4097 mothers were followed in the cohort, continuously recording the pregnancy outcome for those who gave birth during the follow-up period. Among them 48(1.2%) were lost to follow- up and their pregnancy outcome could not be registered. At the end of the follow-up period 102(2.5%) mothers had perinatal death, 45(44.1%) were early neonatal deaths and 57(55.9%) were stillbirths. The perinatal mortality rate was 25.1(95% CI, 20.3–29.9) per 1000 live and stillbirths. The stillbirth rate was 14.1(95% CI, 8.0–17.7) per 1,000 births and early neonatal mortality rate was 11.3 (95% CI, 7.1–14.6) per 1,000 live births. From the total respondents (n = 306) most (74.4%) were mothers, 9.6% were fathers, 5% were grandparents and the rest were siblings or relatives of the index baby.

### Socio- economic and demographic characteristics of the participants

More mothers whose newborns were alive had a primary education than those who lost their newborns. From the participants who live among a household size of two, only two were among the group whose newborns were alive during the time of data collection (Table 1).

**Table 1:** Socio-economic and demographic variables of the participants in West Gojam zone, Ethiopia, Feb 2012(n = 306).

Socio-economic and demographic markers	Cases(n = 102) No (%)	Controls (n = 204) No (%)
**Age of the mother**		
18–24	12(11.8)	21(10.3)
25–29	34 (33.3)	87 (42.6)
30–34	41(40.2)	74 (36.3)
35–40	15(14.7)	22 (10.8)
Mean age ± SD	29.47 ± 4.3	29.07 ± 3.8
**Maternal education**		
Illiterate	99 (97.1)	194 (95.1)
Primary education(1–6) and above	3 (2.9)	10 (4.9)
**Paternal education**		
Illiterate	95 (93.1)	187 (91.7)
Primary education(1–6) and above	7 (6.9)	17 (8.3)
**Maternal occupation**		
Farmer	31 (30.4)	66 (32.4)
House wife	71(69.6)	138 (67.6)
**Household size**		
2	11(10.8)	2 (1)
3–5	37(36.3)	90 (44.1)
6+	54(52.9)	112 (54.9)
**Wealth quintiles**		
Lowest	24 (23.5)	37 (18.1)
Second	14 (13.7)	47 (23)
Middle	22 (21.6)	40 (19.6)
Fourth	18 (17.6)	41 (20.1)
Highest	24 (23.5)	39 (19.1)

### Determinants of perinatal mortality

In the two binary multivariable logistic regression models eight variables were independently associated with perinatal mortality. Newborns who were born in a household of 3–5 (AOR = 0.05, 95% CI 0.01–0.28) and those with more than six (AOR = 0.06, 95% CI 0.01–0.27) had lesser odds of dying during the perinatal period. Newborn babies from families in the second quintile of the wealth index had a lesser odds of facing perinatal mortality (AOR = 0.38, 95% CI 0.16–0.93) as compared to those newborns from households in the lowest quintile (Table 2).

**Table 2:** Socio-economic and demographic factors associated with perinatal mortality in west Gojam zone, Ethiopia Feb 2012(n = 306).

Variables	Cases(n = 102) No (%)	Controls(n = 204) No (%)	Crude OR (95% CI)	Adjusted OR (95% CI)
**Age of the mother**				
18–24	12(11.8)	21(10.3)	1	1
25–29	34 (33.3)	87 (42.6)	0.68 (0.3, 1.54)	0.99 (0.36, 2.67)
30–34	41(40.2)	74 (36.3)	0.97 (0.43, 2.17)	1.63 (0.57, 4.69)
35–40	15(14.7)	22 (10.8)	1.19 (0.45, 3.14)	2.15 (0.63, 7.38)
**Maternal education**				
Illiterate	99 (97.1)	194 (95.1)	1	1
Primary education(1–6) and above	3 (2.9)	10 (4.9)	0.59 (0.16, 2.19)	0.71 (0.1.6, 3.25)
**Paternal education**				
Illiterate	95 (93.1)	187 (91.7)	1	1
Primary education(1–6) and above	7 (6.9)	17 (8.3)	1.12 (0.42, 3.01)	0.67 (0.24, 1.89)
**Maternal occupation**				
Farmer	31 (30.4)	66 (32.4)	1	1
House wife	71(69.6)	138 (67.6)	1.09 (0.66, 1.83)	1.27 (0.67, 4.42)
**Household size**				
2	11(10.8)	2 (1)	1	1
3–5	37(36.3)	90 (44.1)	0.08 (0.02, 0.35)	0.05 (0.01, 0.28)
6+	54(52.9)	112 (54.9)	0.09 (0.02, 0.41)	0.06 (0.01, 0.27)
**Wealth quintiles**				
Lowest	24 (23.5)	37 (18.1)	1	1
Second	14 (13.7)	47 (23)	0.46(0.21, 1.01)	0.38 (0.16, 0.93)
Middle	22 (21.6)	40 (19.6)	0.85(0.41, 1.76)	0.89 (0.38, 2.11)
Fourth	18 (17.6)	41 (20.1)	0.68 (0.32, 1.44)	0.72 (0.29, 1.75)
Highest	24 (23.5)	39 (19.1)	0.95(0.46, 1.96)	0.76 (0.32, 1.77)

Newborns who were born to primiparous mothers had more chance of dying in the perinatal period than those who were born to grand multiparas (AOR = 3.15, 95% CI 1.03–9.60). Similarly, newborns who were born to mothers who had a history of previous perinatal death had an increased odds of perinatal mortality (AOR = 9.55, 95% CI 4.67–19.54). Preterm babies were found to be at an increased risk for perinatal death (AOR = 9.44, 95%CI 1.81–49.22) than term newborns. Home delivery was significantly associated with perinatal mortality (AOR = 0.07, 95% CI 0.02–0.24) (Table 3). When obstetric related variables and possible socioeconomic confounding variables were controlled twins showed three times more odds of dying in the perinatal period than singletons (AOR = 3.46, 95% CI 1.02–11.74).

**Table 3:** Pregnancy, labour and delivery related variables and perinatal mortality in West Gojam zone, Ethiopia Feb 2012(n = 306).

Variables	Cases(n = 102) No (%)	Controls(n = 204) No (%)	Crude OR (95%CI)	Adjusted OR (95%CI)
**Sex of the new born**				
Male	59 (60.8)	116 (56.9)	1.18 (0.72, 1.93)	1.22 (0.64, 2.31)
Female	38 (59.2)	38 (43.1)	1	1
**Number of deliveries**				
One	13 (12.7)	14 (6.9)	1.57 (0.69, 3.56)	3.15 (1.03, 9.60)
2–4	28 (27.5)	87 (42.6)	0.54(0.32, 0.92)	0.99 (0.49, 1.97)
≥5	61 (59.8)	103 (50.5)	1	1
**History of abortion**				
Yes	22 (21.6)	17 (8.3)	3.03 (1.53, 6.00)	2.46 (1.03, 5.86)
No	80 (78.4)	187 (91.7)	1	1
**History of perinatal death**				
Yes	50 (9.8)	20 (6.8)	8.85 (4.84, 16.17)	9.55 (4.67, 19.54)
No	52 (90.2)	184 (93.2)	1	1
Gestational age at delivery				
Preterm (< 37weeks)	14 (13.7)	2 (1)	16.07(3.58, 72.2)	9.44 (1.81, 49.22)
Term (>37weeks)	88 (86.3)	202 (99)	1	1
**Birth spacing**				
< 2years	1 (1)	5 (2.5)	0.37 (0.04, 3.20)	0.23 (0.02, 2.31)
2 years	4 (3.9)	20 (9.8)	0.37 (0.12, 1.11)	0.34 (0.09, 1.32)
> 2years	97 (95.1)	197 (87.7)	1	1
**Sign of pregnancy related complications**				
Yes	12 (11.8)	13 (6.4)	1.96(0.86, 4.46)	1.09(0.36, 3.32)
No	90 (88.2)	191 (93.6)	1	1
**Delivery**				
Singleton	95 (93.1)	199 (97.5)	0.34(0.11, 1.10)	0.30 (0.07, 1.38)
Twin	7 (6.9)	5 (2.5)	1	1
**Place of delivery**				
Home	78 (76.5)	199 (97.5)	0.08 (0.03, 0.22)	0.07 (0.02, 0.24)
Health facility	24 (23.5)	5 (2.5)	1	1
**Mode of delivery**				
Spontaneous vaginal delivery	95 (93.1)	199 (97.5)	0.34 (0.11, 1.10)	0.96 (0.19, 4.72)
Caesarian section	7 (6.9)	5 (2.5)	1	1
**Cord prolapse**				
Yes	5 (4.9)	4 (2)	2.58 (0.68, 9.81)	3.52 (0.66, 18.88)
No	97 (95.1)	200 (98)	1	1

### Causes of perinatal mortality

For ten of the cases (9.8%) there was not enough data to identify the causes of death. For six (5%) of the cases dissimilar diagnosis was assigned to by the two coders. However, the two physicians reached at consensual diagnosis after discussing on the VA data (Table 4).

**Table 4:** Probable causes of stillbirths and early neonatal deaths from a verbal autopsy data in west Gojam zone, Ethiopia Feb 2012(n = 102).

Causes of early neonatal death	No (%)	Causes of Stillbirth	No (%)
Bacterial sepsis	23 (51.1)	Obstructed labour	9 (15.8)
Birth asphyxia	14 (31.1)	Chorioamnionitis	8 (14.0)
Accidental fall	2 (4.4)	Prematurity	7 (12.3)
Birth injury	1 (2.2)	Ante partum hemorrhage	9 (15.8)
Hemorrhagic disease	1 (2.2)	Hypertensive disorder of pregnancy	9 (15.8)
Unspecified hemorrhage	1 (2.2)	Heart disorder	3 (5.3)
Prematurity	1 (2.2)	Bleeding from the umbilicus	1 (1.8)
Suffocation	1 (2.2)	Anemia	2 (3.5)
Unidentified causes	1 (2.2)	Unidentified causes	9 (15.8)
**Total**	**45 (100)**		**57(100)**

## Discussion

In the study area perinatal mortality rate was found to be significantly low as compared to the regional estimates within Ethiopia. But still the rate is considerably high even as compared to African regional standards. Size of the household, household wealth, number of deliveries, previous history of perinatal death and abortion, preterm birth, twin birth and home delivery were significantly associated with perinatal mortality.

In Ethiopia, most facility and population based studies showed perinatal mortality rates which are much higher than what was estimated in this study. According to the EDHS 2011 report the overall National perinatal mortality rate was 46 per 1000 live and stillbirths [3]. And the estimate for Amhara regional state was 55 per 1000 live and stillbirths [3]. Similarly a study conducted in Jimma Hospital on perinatal mortality rate and its trend from 1990–1999 indicated an average perinatal mortality rate of 138 per 1000 pregnancies [11].

Perinatal mortality rate differences between the finding of this study and the EDHS can be explained by the variation in the scope of the studies and the level that the results were inferred for. In the EDHS, the number and selection procedure of clusters were done with the intention of generating rates that are representative at the regional and the national levels [3]. Therefore, the existing variation in perinatal mortality might have partly resulted from the difference in the scope and level of the two studies.

Hospital based studies have a tendency to overestimate deaths unless all deliveries of the given source population take place in health facilities [1]. Especially, perinatal mortality is highly prone for overestimation in hospital based studies. Most laboring mothers who visit hospitals are referred from the nearby primary health care facilities for a better care, when the condition of the mother or the newborn is subjected for referral. This may increase the likelihood of perinatal death in health facilities, taking in to account the tendency to delay before reaching hospitals. So, these conditions may explain the significant difference in perinatal mortality rates of hospital based studies and our study.

Perinatal mortality is one of the cardinal indicators of obstetric care quality. The MaNHEP intervention that was designed to increase coverage and enhance quality of prenatal, intranatal and post natal care can also explain the lower perinatal mortality rate in the study area than the regional and national perinatal mortality estimates.

### Wealth and perinatal mortality

Parents in the second wealth quintile had a lesser risk of losing their baby for perinatal death as compared to those in the first quintile. Existing evidences strongly suggest that poverty favors the occurrence of perinatal mortality [12]. Unfavorable living condition and lack of money to cover for medical expenses can be considered as the links between poverty and increased perinatal death. Even in settings where the service might be affordable there is reluctance to seek care due to fear of high service cost. But those parents in the third and higher quintiles didn’t show a significant difference in the risk of perinatal death, compared to those in the lowest category. This absence in association can be attributed to the nature of the analysis, wealth index shows the relative wealth difference among the households that are involved in the study, not the absolute wealth ownership [13]. If a study is conducted in poor community, like the case of our study, the effect of wealth may not stand out since the comparison is merely among the poor.

### Parity and household size

In this study newborns who were born to mothers who gave birth to two or more children prior to the last delivery showed a better chance of surviving the perinatal period when compared to the first babies. Primigravid mothers are at an increased risk for pregnancy, labour and delivery related complications, which require emergency obstetric care [14]. Unfortunately, only part of emergency obstetric care service package is provided at health posts and health centers. The nearest hospital where this service package can be fully obtained is Bahirdar Hospital, which is on average 90 kilometers away from the study area [15]. So, primigravids have low chance of obtaining the service before the complications happen, especially since most deliveries are first attempted at home [16]. This result is consistent with studies from other parts of Ethiopia, Uganda and Kenya [5, 14 and 17], indicating increased risk of perinatal mortality among first born babies. This may also explain the low risk of perinatal death among households with a size of at least three and above as compared to those with a size of two. Most families with a size of two could possibly be newly married couples who had their first baby. In their culture it is customary for most women to get pregnant with their first baby in the first year of marriage [16].

### Previous history of perinatal mortality

After adjusting for possible obstetric and health care service related confounders, women with a history previous perinatal mortality had nine times more risk of losing their newborns to perinatal death during their last pregnancy. This association could result from the improper newborn handling practices which prevail in the community for ages [16]. These practices are likely to affect most of the newborns in the particular family, if not all, as far as the practices are still exercised. Furthermore, when a mother loses a new born soon after birth there is a desire to replace the lost baby immediately [16]. The need to have another baby sooner, leads to narrow birth spacing which increases the risk of newborn death. Population based prospective studies in Tanzania and a population based prospective cohort study in six countries in West Africa showed a similar strong association between previous history of child mortality and perinatal mortality during the last pregnancy [18, 19].

### History of abortion

In our study abortion was considered as the termination of pregnancy before the 28^th^ week of gestation. Mothers with no previous history of abortion had a lower risk of losing their newborn for perinatal mortality as compared to those who had a history of abortion. Several studies indicated that repeated abortion is associated with adverse pregnancy outcomes such as bleeding in the first trimester, preterm birth, low birth weight and perinatal mortality [20,21, 22]. In addition, the low ANC service utilization deters mothers from preventing possible risk factors predisposing to abortion and other adverse pregnancy outcomes.

### Twin birth and perinatal mortality

The pregnancy, labour and delivery process for twins is prone for risks like prolonged second stage, compound presentation leading to trauma, antepartum hemorrhage and others [23]. These factors are among the known predictors of stillbirth and early neonatal mortality [23]. The ANC service provided by HEWs that have limited experience, training, and insufficient equipments to identify and deal with twin pregnancy in advance is one of the factors for the inability to prevent the complications related to twin pregnancy. In addition, the limited institutional delivery service utilization, and questionable capability of healthcare providers to deal with obstetric complications might have increased the risk of mortality among twins than singletons.

### Gestational age at delivery

Preterm birth is associated with anatomic and physiologic underdevelopments of all the systems of the baby which may aggravate the risk of perinatal death. Pulmonary immaturity, respiratory distress syndrome and vulnerability to infections due to the underdeveloped immune system are among the leading causes of death for preterm newborns [24]. Preterm birth is one of the known risk factors for perinatal mortality even in settings where advanced neonatal care is available [25]. In rural communities where advanced neonatal care is absent the chance of surviving the physiological underdevelopments is very low.

### Place of delivery

Many studies revealed a strong association between perinatal mortality and home delivery [26, 27]. But our study showed that home delivery is not associated with increased risk of perinatal mortality. The study area has a low institutional delivery rate, which was not more than 10% according to the 2011 DHS report [3]. Therefore, probably most of the women who visit health facilities for delivery care attempt home delivery first till complications are eminent. In addition, as part of the intervention a number of birth attendants were trained to assist HEWs with providing clean and safe delivery at home. So, even if there were home deliveries the birth could possibly be attended by HEWs, who had additional training on labour and delivery care and assisted by trained TBAs. But we need to point out that who attended the deliveries had to be asked to have a better clarity regarding the association between place of delivery and perinatal death.

### Causes of perinatal mortality

The verbal autopsy result indicated that the leading causes of perinatal death were infection, birth asphyxia, antepartum hemorrhage, hypertensive disorders of pregnancy and obstructed labour. The high prevalence of causes like asphyxia, obstructed labour and infections indicates the limited access to skilled delivery care. In the study area, more than ninety percent of the births took place at home. The extended time the mother spends with obstructed labour before going to health facilities, while attempting home delivery, predisposes the baby to distress leading to asphyxia. But distance of the only hospital which can fully provide emergency obstetric care and the reluctance to seek institutionalized delivery care contributed to the occurrence of perinatal death secondary to the mentioned preventable obstetric complications. A longitudinal study in Bangladesh clearly showed that improved delivery care and increased number of institutional deliveries can significantly reduce most of the mentioned causes of early neonatal death [28].

### Limitations of the study

The MaNHEP intervention kebeles, where this study was conducted, were purposively selected based on the project criteria for the intervention. So, these districts have a better proximity to health care facilities and the referral hospital. Also, perinatal deaths were categorized as a still birth or early neonatal death only based on the respondents subjective feedback with no objective measurement. This may cause misclassification of still births and early neonatal deaths, specially for newborns who died right after birth.

## Conclusion

High perinatal mortality rate in the study area signified the need for sustainable community based interventions. The increased risk of losing newborns for perinatal death among Primipara mothers and mothers with a previous history of perinatal death and abortion points out the need for enhancing the quality and utilization ANC service. Birth asphyxia, infections, obstructed labour, preterm birth, antepartum hemorrhage and hypertensive disorders of pregnancy calls for quality delivery care. Improving socioeconomic status of the community also helps to reduce perinatal death associated with poverty. Finally, the effect of home delivery on perinatal death should be further studied.
